# Detecting the Orbital Angular Momentum of Electro-Magnetic Waves Using Virtual Rotational Antenna

**DOI:** 10.1038/s41598-017-04313-4

**Published:** 2017-07-04

**Authors:** Chao ZHANG, Lu MA

**Affiliations:** Labs of Avionics, School of Aerospace Engineering, Tsinghua University, Beijing, 100084 P. R. China

## Abstract

Orbital Angular Momentum (OAM) is a typical spatial mode of an Electro-Magnetic (EM) wave. Correctly detecting the OAM mode is fundamental and of foremost importance when applying the phenomenon to wireless transmission in free space. It is found that when rotating an OAM wave, a rotational Doppler shift that is proportional to the rotation speed and the OAM mode number can be observed. This property can be used for OAM detection, i.e., different OAM modes are identified by measuring the corresponding rotational Doppler frequency shifts. In previous work, this method was implemented by mechanically rotating the OAM wave, resulting in a small frequency shift. Since the high-speed mechanical rotation is hard to manufacture in a real system, it brings limitations to the bandwidth for each OAM wave. In this paper, we report on an OAM mode detection method based on digitally rotating a virtual antenna. The transmitter and receiver are physically fixed, but the Virtual Rotational Antenna (VRA) is obtained by interpolating the signals received from transverse-mounted receiving antennas. A large rotational Doppler shift occurs as a consequence of using digital processing, resulting in more capability for wideband wireless data transmission with the larger shifted frequency.

## Introduction

As first revealed by Allen in an optical vortex (i.e., a light beam that has an azimuthally dependent phase)^1^, Orbital Angular Momentum (OAM) has since been widely studied in optics^2–5^. OAM was always considered a spatial mode basis in Space Division Multiplexing (SDM)^6^ in optics in addition to many other bases^7, 8^. The concept of OAM was then extended to radio frequencies by Thidé^9^ and subsequently studied by other researchers^10–13^. It is of fundamental importance to correctly detect the OAM mode of an Electro-Magnetic (EM) wave when applying it to wireless transmission in free space. The Poynting vector of an OAM wave traces a helical phase along the propagation axis, and there is also an angle between the incident beam and the receiver. When mechanically rotating the OAM wave, a frequency shift proportional to the speed of rotation and the OAM mode number can be observed. This is the so-called rotational Doppler Effect as revealed in optics^14–17^ and millimetre wave experiments^18^. It is a different phenomenon than polarization rotation as revealed by Li^17^. In fact, this rotational Doppler Effect is just one example of Doppler velocimetry where the angle between the incident beam and the detector is a skew angle. This angle increases proportionally with the OAM mode. This effect can be used to detect the OAM mode of EM waves, i.e., different OAM modes are identified by measuring the corresponding rotational Doppler frequency shifts.

In previous work, the method used was to mechanically rotating the OAM mode at the transmitting side by, for example, rotating a Spiral Phase Plate (SPP) used to generate that OAM mode^19^. This mechanical rotation of the transmitter resulted in the expected rotational frequency shift upon OAM detection. However, the rotation speed is limited mechanically and it is hard to manufacture and implement with high speeds, so the rotational Doppler shift is not large, e.g., usually less than several hundred Hz. This limitation becomes notable, especially in the case of wireless data transmission in free space. As described in previous work^18^, the frequency shift can be considered as the carrier bandwidth of each OAM wave. A small frequency shift, therefore, leads to a small carrier bandwidth which limits the data transmission rate (see Fig. S3 in Supplementary Information). Moreover, from the perspective of OAM measurements, higher frequency shifts result in higher resolution frequency detection, which implies accurate OAM mode detection.

Note that the frequency shift is caused by the rotation of the OAM wave relative to the detection point. It will also work when rotating the detecting antenna azimuthally with respect to the beam axis of the OAM wave while keeping the OAM wave stationary. In this case, the rotational Doppler frequency shift can also be measured. However, due to the divergence of the doughnut-shaped beam, the rotating radius required at the receiving antenna (or antenna array) will increase with the distance. For instance, for a 10 GHz radio wave with an OAM mode *l* = 1, a half beam divergence angle of 0.1 rad will lead to a doughnut-shaped beam with a radius of 1 km at a distance of 10 km. The divergence of the doughnut-shaped beam implies that it is impractical to physically rotate the receiving antenna azimuthally using mechanical means.

Here, we demonstrate an equivalent rotation method implemented digitally at the receiving side using a Virtual Rotational Antenna (VRA). This is done by interpolating the signals received from transverse-deployed receiving antennas. Specifically taking two receiving antennas as an example, the OAM wave is first sampled by these two antennas which have been transversely deployed across the doughnut-shaped beam. The measured signals are then digitally processed using an interpolation algorithm based on the channel correlation between the antennas to generate the computed signal of a VRA moving between the two real fixed antennas. According to the rotational Doppler Effect, if the VRA rotates with a certain rotational speed, a frequency shift in the interpolated signal occurs and the corresponding OAM mode can be detected and identified. A similar concept of interpolation using an antenna array was first introduced to eliminate the Doppler Effects in wireless digital TV broadcasting^19^ and then extended to Air-to-Ground (A/G) MIMO communications^20^. Because of this, we built a novel interpolation scheme to realize the VRA. Although this OAM detection method is verified using a sinusoidal signal without modulated symbols, it can also detect the data encoded on the OAM by restraining the rotation period of the virtual antenna within the symbol period (see Fig. S4 in Supplementary Information). Moreover, it should be emphasized that, in this paper, only the OAM detection method for single mode is studied and highlighted, leaving the simultaneous detection of multiple OAM modes in multiplexing for future research.

## Results

### Method for generating the VRA

**Figure 1:**
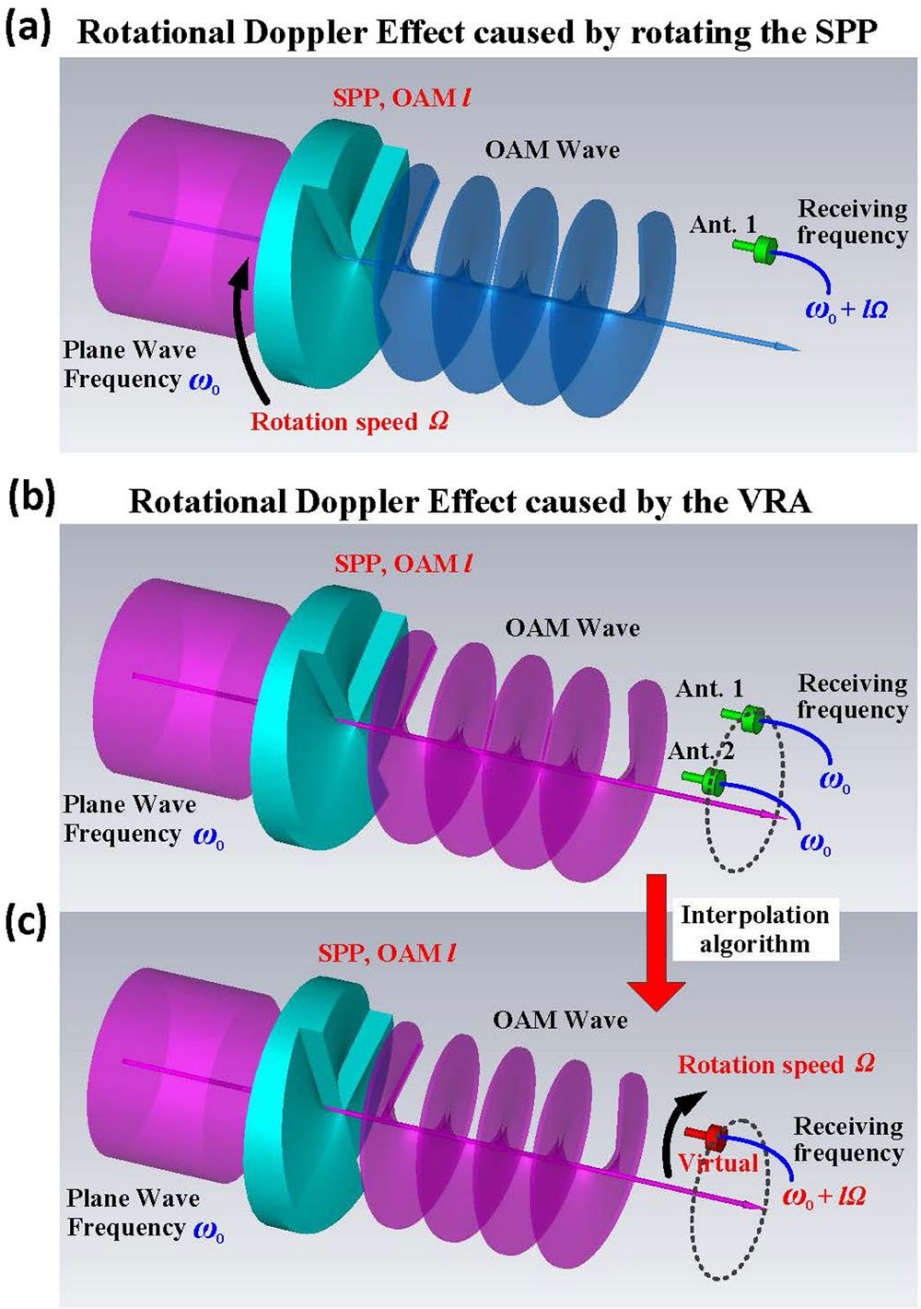
Doppler Effect caused by the VRA. (**a**) Detecting the OAM mode by rotating the SPP at the transmitter. Rotating the SPP of mode *l* with speed *Ω*, a frequency shift *l* *Ω* can be detected by a fixed receiving antenna. (**b**) Receiving OAM wave using two fixed antennas. Two transverse-deployed antennas are used to receive the OAM wave generated by a fixed SPP. The frequency detected by each antenna is the same as that of the transmitter. No frequency shift arises. (**c**) Receiving OAM wave using the VRA. By weighting and adding the two received signals, an interpolated signal is generated. The phase of the interpolated signal gradually varies between one antenna and the other. This performs similarly to a virtual antenna, rotating from one antenna to the other. Due to the rotational Doppler Effect, a frequency shift can be detected in the interpolated signal.

An illustration of the VRA is shown in Fig. 1. In our previous work (Fig. 1a), the method for detecting the OAM mode was implemented by rotating the SPP at the transmitter and detecting the frequency shift at the receiver. A frequency shift of *l* *Ω* will be detected when rotating the SPP of mode *l* with rotational speed *Ω*. As discussed in the introduction, this frequency shift is caused by the relative rotation between the OAM wave and the detector. A frequency shift can also be generated by rotating the receiving antenna while keeping the SPP stationary at the transmitter. Here, the receiving antenna is rotated digitally (i.e., subjected to virtual rotation) instead of being mechanically rotated. This is done by interpolating the signals received from two fixed transverse-deployed antennas (Fig. 1b). In the interpolation, the two received signals are weighted and added. By setting the weighting coefficients to vary with time, the phase of the interpolated signal can be set to vary gradually between the signal of one antenna and the signal of the other antenna. This performs as though an antenna exists between the two antennas and moves from one antenna to the other (Fig. 1c). Due to the rotational Doppler Effect, a frequency shift can be detected in the interpolated signal. This frequency shift will be different when transmitting different OAM modes and is proportional to the rotational speed *Ω* and the OAM mode number *l*, which is expressed as *l* *Ω*. In this case, the OAM mode can be detected by measuring the frequency shift of the interpolated signal. Since the virtual antenna is generated digitally using Digital Signal Processing (DSP) methods, its rotational speed *Ω* can be increased significantly compared to the mechanical rotation. Larger frequency shifts can thus be obtained which will benefit the OAM mode detection accuracy and bandwidth requirements of wireless transmission. Note that since the frequency detected at any reception point is the same as that of the transmitter, there are no new frequencies introduced in the transmission. The resultant frequency shift is just a result of interpolation and signal processing.

### Mathematical model of the interpolation

**Figure 2:**
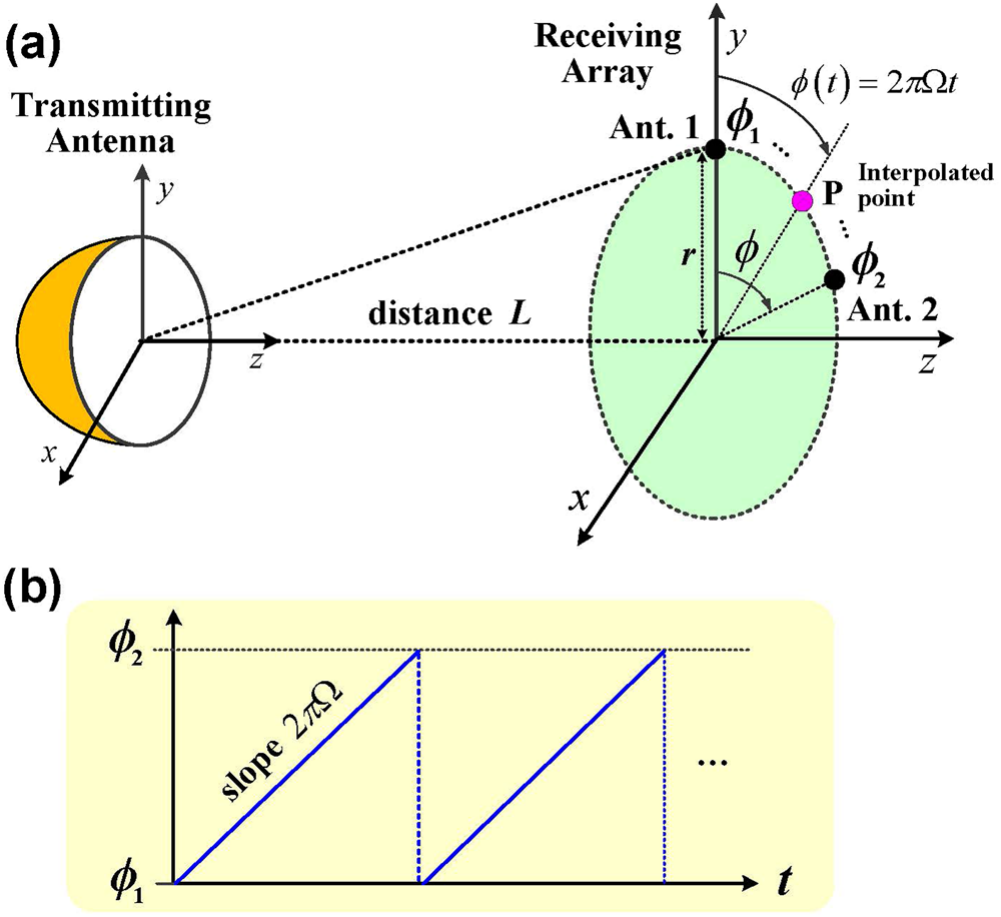
Interpolation of the antenna array signals. (**a**) Schematic of the interpolation algorithm. The OAM wave is detected using two antennas deployed transversely across the doughnut-shaped beam. These two antenna signals are multiplied by weighting coefficients and added together. This interpolated signal has the characteristics of a signal that would be received by an antenna positioned at a point between the two antennas. By varying the weighting coefficients with time, the location of the interpolated point can be made to move from one antenna to the other. (**b**) The rotation angle of the interpolated point P which rotates between Ant. 1 and Ant. 2 with speed *Ω*.

The mathematical model of the interpolation between two antennas is shown in Fig. 2a. Consider the case of two receiving antennas. The vector based on the antenna signals at time *t* can be expressed as

1 $${\bf{r}}(t)={[{r}_{1}(t),{r}_{2}(t)]}^{{\rm{T}}}$$

where *r*_1_(*t*) is the signal received from antenna 1 (Ant. 1), and *r*_2_(*t*) is the signal received from antenna 2 (Ant. 2).

Denoting the normalized expectation of a random variable *x* by *E*[*x*], the autocorrelation of the received signal can be derived based on Eq. (1) as

2 $${\bf{R}}(t)=E[{\bf{r}}(t)\cdot {{\bf{r}}}^{{\rm{H}}}(t)]=[\begin{array}{cc}1 & {J}_{0}(l({\varphi }_{2}-{\varphi }_{1}))\\ {J}_{0}(l({\varphi }_{1}-{\varphi }_{2})) & 1\end{array}]$$

﻿where *J*_0_(*x*) denotes t﻿he zero-order Bessel function of *x*. For the case of a rotating receiving antenna, the interpolated point should rotate azimuthally between the receiving antennas, i.e., the position of the interpolated point should rotate from Ant. 1 with the rotation angle of *ϕ*_1_ to Ant. 2 with the rotation angle of *ϕ*_2_ periodically (Fig. 2b). Denoting the rotational speed of the interpolated point by *Ω*, the rotation angle of the interpolated point P can be expressed as

3 $$\varphi =2\pi {\Omega }t\in [{\varphi }_{1},{\varphi }_{2}]$$

Thus, the cross-correlation matrix between the interpolated signal at the point P (denoted by *r*(*Ω*, *t*)) and the received signals can be derived as

4 $${\bf{P}}{\boldsymbol{(}}{\Omega }{,}{t}{)}=E[{\bf{r}}(t)\cdot {r}^{\ast }({\Omega },t)]=[{J}_{0}(-2\pi l{\Omega }t),{J}_{0}(l(\varphi -2\pi {\Omega }t))]$$

where “*” denotes conjugate. By interpolating to satisfy the Minimum Mean Square Error (MMSE)^19, 20^, the weighting coefficient **w** for the received vector can be expressed as

5 $${\bf{w}}({\Omega },t)={\bf{R}}{(t)}^{-1}{\bf{P}}({\Omega },t)$$

Therefore, the optimal estimate of the interpolated signal can be expressed as

6 $${r}_{{\rm{V}}{\rm{R}}{\rm{A}}}({\Omega },t)={{\bf{w}}}^{{\rm{T}}}({\Omega },t){\bf{r}}(t)={{\bf{P}}}^{{\rm{T}}}({\Omega },t){\bf{R}}(t{)}^{-{\rm{T}}}{\bf{r}}(t)$$

Due to the rotational Doppler Effect, the frequency shift can be detected from the interpolated signal *r*_VRA_(*Ω*, *t*). Although two receiving antennas are used here, the concept can be extended to an antenna array with more elements^19, 20^ (see Fig. S5 in Supplementary Information).

### Experiment using VRA for OAM detection

**Figure 3:**
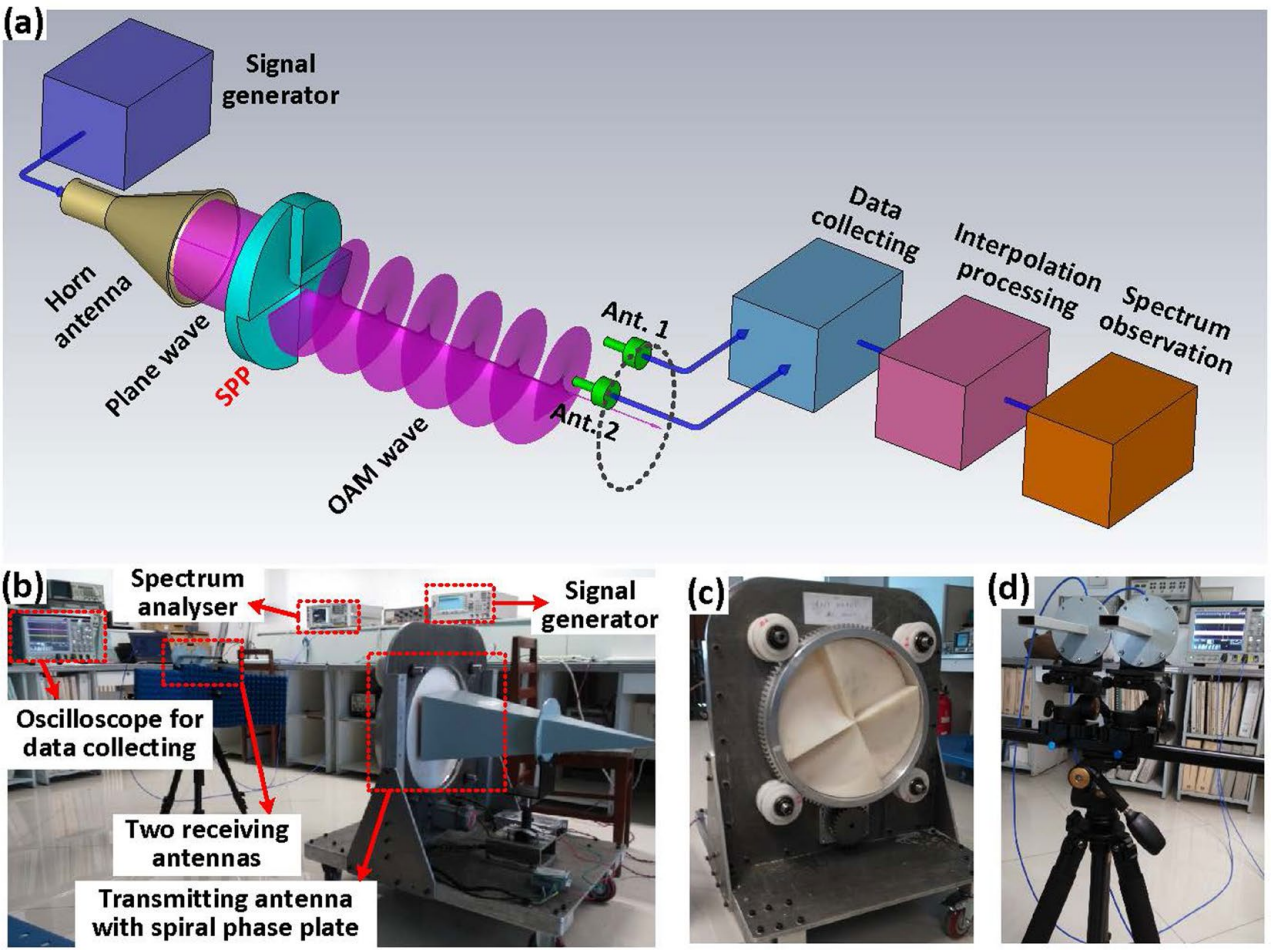
Structure of the experimental system. (**a**) Transceiver structure for detecting the OAM mode using the VRA method. An EM beam is generated by a horn antenna and then passes through the SPP, which produces an OAM wave with mode number *l*. At the receiving side, the OAM wave is received by two antennas, which are deployed transversely across the doughnut-shaped beam, and then digitally sampled. The sampled data are input to a computer where the interpolation is performed using MATLAB. Afterwards, the interpolated data are output to a spectrum analyser (Agilent E4446A) for observation. (**b**) OAM mode detection experiment conducted in the laboratory. (**c**) Transmitting antenna fabricated by placing the SPP in front of a horn antenna. (**d**) Waveguide antennas used to receive the signals.

The design of an experiment using the VRA to detect the OAM mode is shown in Fig. 3a and its implementation is shown in Fig. 3b. As shown in Fig. 3a, a sinusoidal signal generated by a signal generator is fed to the transmitting antenna. The radiated beam passes through the SPP with a specific OAM mode. At the receiving side, the signals are received by two transverse-deployed antennas and collected by a data collecting device. The sampled data are input into a computer for signal processing. In the computer, a VRA with a specific speed of rotation is generated by the interpolation algorithm and used to convert signals with different OAM modes into signals with different frequencies. By calculating the frequency shift of the interpolated signal, the OAM mode can be detected and identified. Based on this design, the experiment conducted is shown in Fig. 3b.

In the experiment, a 10 GHz sinusoidal signal was generated by an Agilent E8257C signal generator. The OAM antenna is a horn antenna with 25 dB gain and an SPP with a specific OAM mode (e.g., OAM mode number *l* = 4, the height difference is ∆*h* = *l* *λ*/(*n* − 1) = 57.7 mm, where *l* is the OAM mode, *λ* is the wavelength, and *n* is the index of refraction of the SPP). Since the novelty of this paper is to demonstrate an OAM detection method, only one OAM mode is required to be generated for each SPP, despite the fact that an SPP can generate not only one OAM mode but also a superposition of OAM modes.

**Figure 4:**
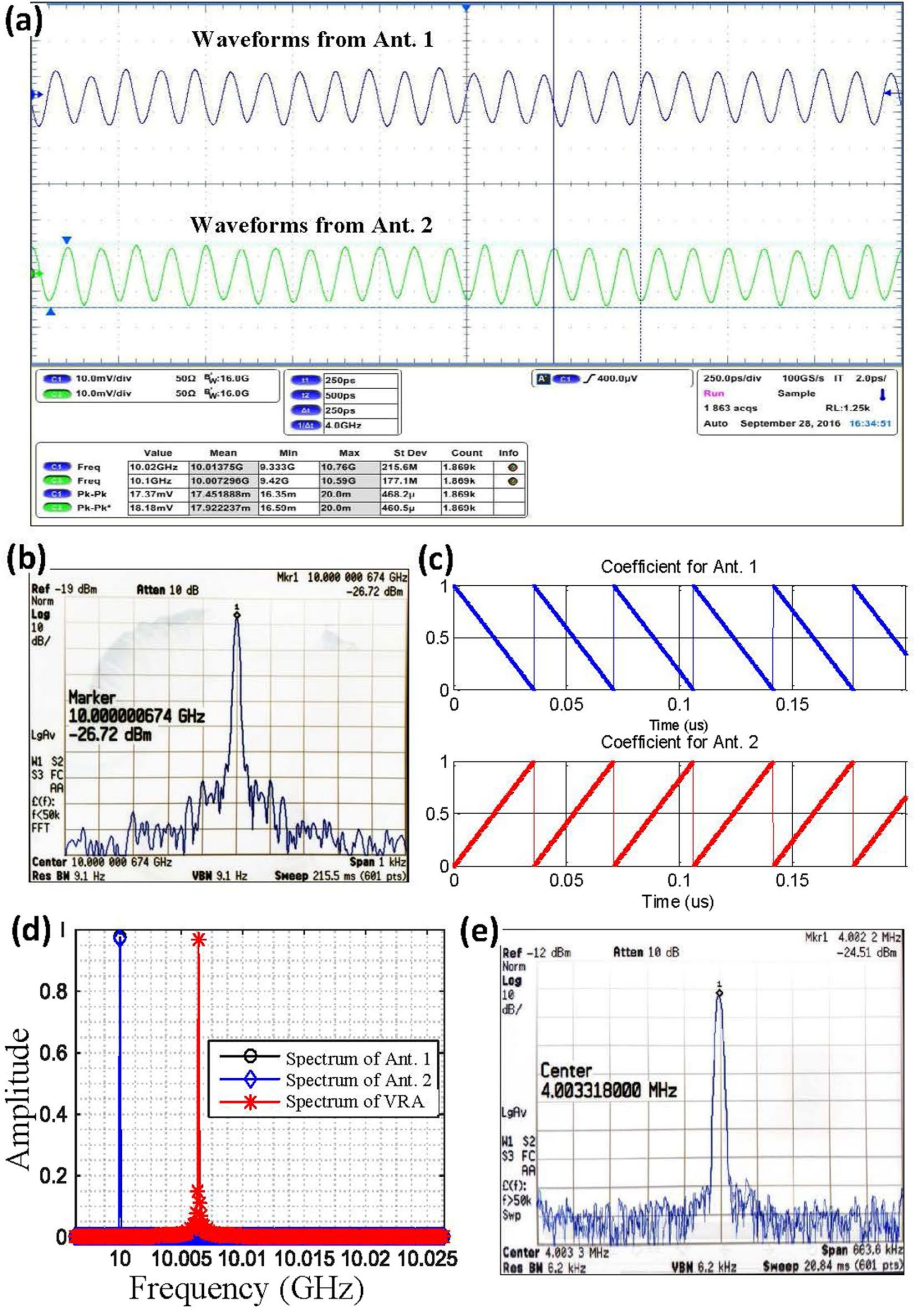
Signals and spectrum observed for OAM mode detection using the VRA. (**a**) Signals received from two antennas. (**b**) Observed spectrum of the signal received from one antenna. (**c**) Weighting coefficients used for interpolation in the experiment. (**d**) Frequency shift detection of the interpolated signal. (**e**) Observed spectrum of the interpolated signal exported from the computer. The interpolated signals were down-converted to zero intermediate frequency by mixing with a carrier signal of 10 GHz frequency and digitally filtered using a low-pass filter. The down-converted signals were then exported from the computer to a signal processing board for data playback. A spectrum analyser was used to observe the spectrum (see Fig. S8 in Supplementary Information).

At the receiving side, two waveguide antennas are deployed transversely across the doughnut-shaped beam to receive the signals. These signals are sampled and recorded by a Tektronix oscilloscope MSO/DPO70000 with 16 GHz bandwidth and 100 GS/s sampling rate (see Fig. 4a for the sampled waveforms). For the signal received from each antenna, the spectrum is observed in Fig. 4b. A Dell Precision T7610 computer is used for interpolation processing. In the computer, a rotation method referred to as VRA is digitally performed with a speed *Ω* = 10^6^ r/s to convert signals with different OAM modes into signals with different frequencies. The weighting coefficients used for the interpolation in the experiment are shown in Fig. 4c. The weighting coefficient for the signal from Ant. 1 decreases linearly with time, whereas the weighting coefficient for the signal from Ant. 2 increases linearly. In this case, the location of the interpolated point will rotate from Ant. 1 to Ant. 2. When transmitting an OAM wave with mode *l* = 4 and setting the speed of rotation of the interpolated point to be *Ω* = 10^6^ r/s, a frequency shift of 4 MHz is detected in the interpolated signal (Fig. 4d). During the experiment, the SPP is stationary. However, for validation, the SPP was rotated mechanically with a speed of *n* = 2.5 r/s to verify that the expected OAM mode was generated at the transmitter. According to our previous work conducted with millimetre-waves^18^ and Courtial, J. with an optical beam^15, 16^, only when a frequency shift of Δ*f* = *ln* (Hz) is observed, is the expected OAM mode generated at the transmitting side (see Fig. S6 in Supplementary Information).

**Figure 5:**
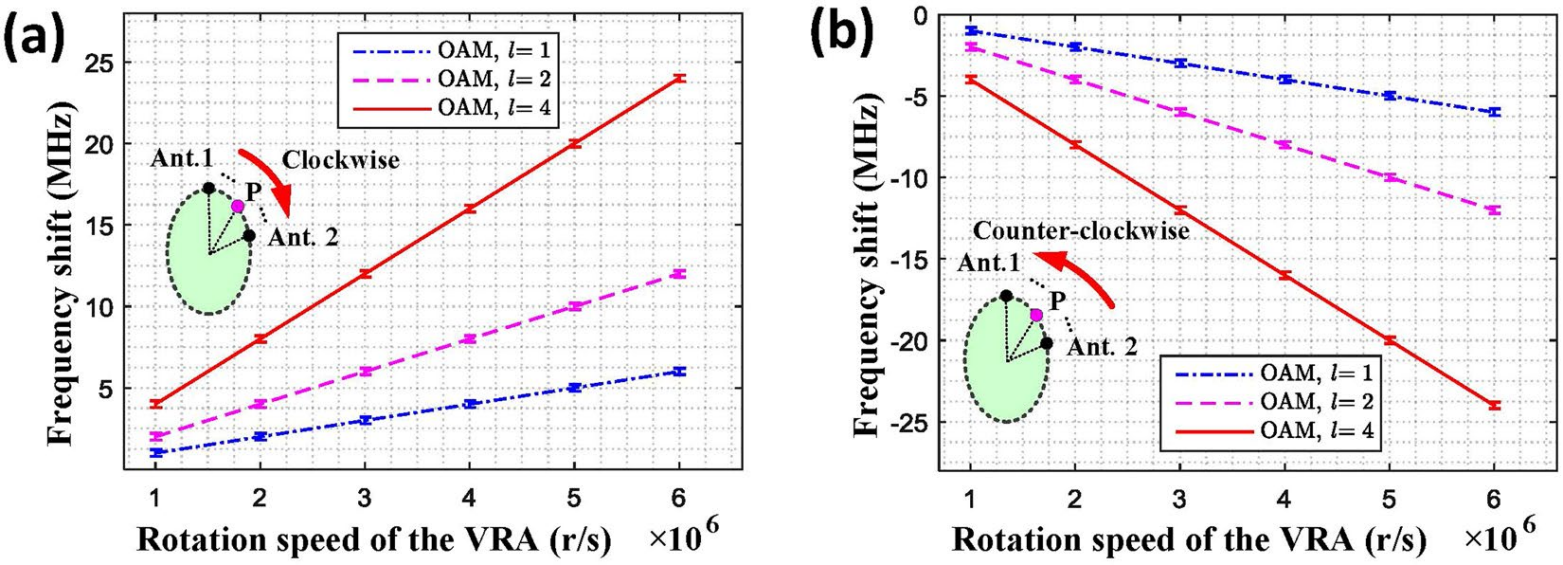
Frequency shift detection for different OAM modes and different rotation speeds. (**a**) Frequency shift detected when the virtual antenna rotates along the positive direction of the OAM mode, i.e., the clockwise direction. (**b**) Frequency shift detected when the virtual antenna rotates along the negative direction of the OAM mode, i.e., the counter-clockwise direction.

Based on this experiment, the frequency shifts introduced by the VRA with different rotation speeds were computed and plotted. A positive frequency shift arises when the VRA rotates (or moves) along the positive direction of the OAM mode (Fig. 5a), while a negative frequency shift arises when the VRA rotates (or moves) along the negative direction of the OAM mode (Fig. 5b). Here, the positive direction of OAM mode is defined by the direction where the phase surface of the OAM beam varies from 0 to 2π azimuthally, and the negative direction of OAM mode is defined by the direction where the phase surface of the OAM beam varies from 2π to 0 azimuthally.

As with the method of detecting the OAM mode by rotating the SPP mechanically at the transmitter^18^, frequency shifts can be detected by rotating the receiving antenna digitally using the VRA method, making it possible to detect the OAM mode using fixed receiving antennas or an antenna array. Compared with the OAM detection method where the SPP is mechanically rotated in front of the transmitter, the rotation speed *Ω* of the interpolated antenna can have a much larger value using the VRA method. However, there is still a limitation. The frequency shift must be greater than the frequency resolution^21^ and smaller than the Nyquist sampling rate^22^. For this, the upper limit of the rotation speed of the VRA is restricted by the sampling rate *f*_s_ and the antenna interval *d*, i.e., *Ω* ≤ (*d*/2π*r*)*f*_*s*_. Here, *r* is the distance from the receiving antenna to the propagation axis of the beam as shown in Fig. 2(a), and *d* is the azimuthal distance between the antennas. The lower limit of the rotation speed is restricted by the frequency resolution *f*_*r*_, i.e., *Ω* ≥ *f*_*r*_. Therefore, the limits of the rotation speed are

7 $${f}_{r}\le {\Omega }\le \frac{d}{2\pi r}{f}_{s}$$

Substituting our experimental parameters into Equation (7), the rotation speed *Ω* varies from 1 r/s to approximately 10^9^r/s. By rotating the receiving antenna digitally using VRA, the frequency shift Δ*f* caused by the rotational Doppler Effect is derived to be Δ*f* = *l* *Ω*.

In summary, an OAM mode detection method implemented by digitally rotating a receiving antenna with VRA has been proposed. As long as the frequency shift of the rotational Doppler Effect is detected, the OAM mode can be detected and identified. Compared with our previous work based on detecting the OAM mode by mechanically rotating the SPP at the transmitter, a larger frequency shift can be detected using fixed antennas and is more easily implemented for wideband wireless data transmission in free space.

## Discussion

As in previous work^18^, by transforming signals with different OAM modes into signals with different frequency shifts based on the rotational Doppler Effect, OAM detection can be performed in the frequency domain rather than the space domain. Receiving only part of the beam using an antenna array is sufficient for OAM detection, and the antennas can be closely spaced. There is no need to receive all the energy in the doughnut-shaped field in the surrounding space. This makes it convenient to measure the OAM mode for free space transmission. The only limitation that may restrict the transmission distance is the power loss caused by beam divergence. This can be partly addressed using a reflector^11, 23^ or lens^18^.

The parameter used for OAM detection in this paper is the rate of variation of the phase gradient, i.e., the frequency shift. This is not the phase gradient referred to by Mohammadi^24^. The phase gradient between the receiving antennas can be very small (unless the interval between the receiving antennas is drastically increased), but the phase variation rate can be very high. Although the single point receiving method can also be used for OAM detection^24^, it is a far-field approximation method and only works well when the beam divergence is small. The transverse component of the electric field must also be well aligned and match the polarization of the field.

In the traditional co-axial receiving scheme similar to the optical transmission, different OAM waves are received in the doughnut-shaped ring perpendicular to the propagation direction. Then, the signals with different OAM modes are orthogonal to each other in the space domain. However, in this paper, the signal of only a quite small portion of the ring is received, which results in the non-orthogonal of the signals in the space domain. In order to maintain the orthogonal of signals with different OAM waves, it is necessary to transform signals to other domain that is easy for detection and identification. The idea of this paper is to transform signals with different OAM modes to the signals in the frequency domain to maintain the orthogonal, i.e., with different frequency shifts. Our proposed scheme has already been verified with the experiment of single mode detection. However, it does not mean that the frequency domain is optimal for simultaneous detection of multiple OAM modes in multiplexing. In this case, new transform domain and method are required to transform signals and maintain the orthogonal. Due to the additive property of the EM waves, the recorded experiment data for single mode detection can also be utilized for further analysis of the simultaneous detection and new transform domain identification in future research.

The last but not the least, the transmitter and receiver do not physically rotate in our proposed scheme. The OAM detection with VRA is expected to be carried out only in the direct-path transmission channel. Multipath will introduce intra-channel and inter-channel crosstalk for signals^25^. This crosstalk can be considered as the interference which will affect the phases and amplitudes of the received signals, and then affect the detection accuracy. However, it does not absolutely hold in all OAM detection schemes. In previous work with physically rotational SPP in the transmiter^18^, the frequency shifts of the multipath signals can also be detected for any fixed receiving point in the space. Thus, in this case, rather than interference, the multipath signals can benefit the transmission performance by employing the processing methods in the wireless transmission such as RAKE receivers^26, 27^.

## Methods

In our experiments, we used SPPs with three OAM modes, i.e., *l* = 1, *l* = 2 and *l* = 4, to generate the OAM waves, and a standard horn antenna with 25 dB gain and a centre frequency of 10 GHz. Two waveguide antennas deployed transversely were used to receive the OAM waves, and a high-speed sampling oscilloscope (Tektronix MSO/DPO70000) was used to collect the data. To verify whether the rotational Doppler Effect can be detected by a VRA, the sampled data were then exported to a computer to perform the interpolation. The frequency detected in the interpolated signals is composed of the carrier frequency and the Doppler frequency shift. By filtering out the carrier frequency, the frequency shift was detected. The down-converted signal was exported to a development board for data playback, and a spectrum analyser was used to observe the spectrum. Meanwhile, in order to guarantee the two receiving antennas were positioned within the doughnut-shaped beam to detect maximal energy, a two-dimensional (2D) adjustable support was used to adjust the positions of the antennas.

## Electronic supplementary material

Supplementary Information
