# Supplementary material for: Detecting the Orbital Angular Momentum of Electro-Magnetic Waves Using Virtual Rotational Antenna
Source: Sci Rep. 2017 Jul 4;7:4585. doi: 10.1038/s41598-017-04313-4 (PMC5496886; doi:10.1038/s41598-017-04313-4)
Supplement: Supplementary file 1 — Supplementary Information [file 41598_2017_4313_MOESM1_ESM.pdf]

Supplementary Information

Detecting the Orbital Angular Momentum of Electro-Magnetic Waves Using Virtual Rotational Antenna

Chao ZHANG*, Lu MA

Labs of Avionics, School of Aerospace Engineering,
Tsinghua University, Beijing, 100084, P. R. China.
*To whom correspondence should be addressed; E-mail: zhangchao@tsinghua.edu.cn

Measurement setup

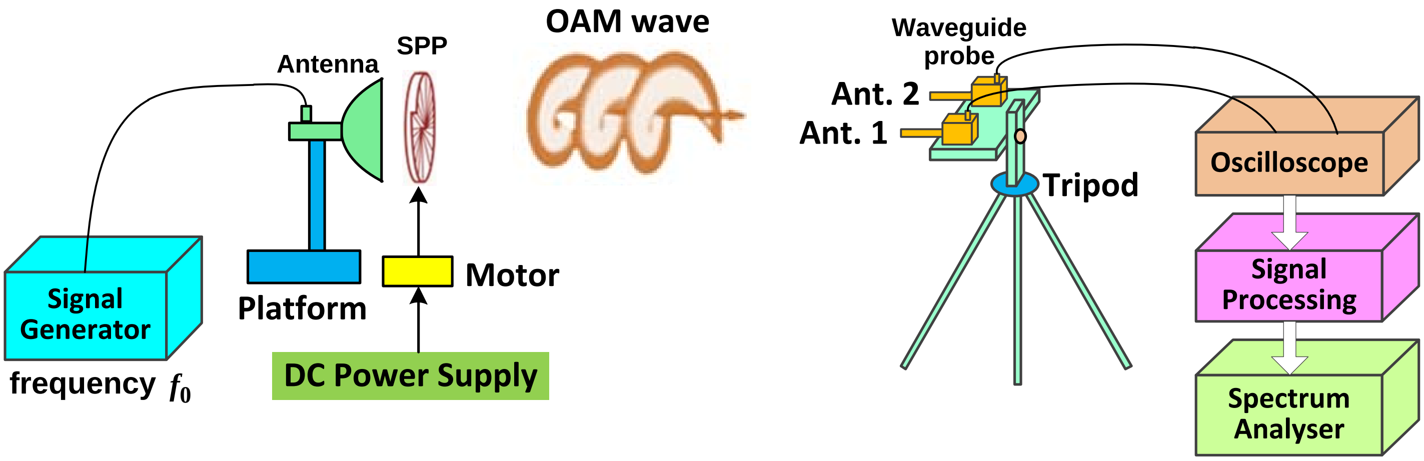

Figure|S1. Experimental setup for the OAM wave received by a Virtual Rotational Antenna (VRA). A sinusoidal signal with a specified frequency is generated by the signal generator, and then fed into a standard horn antenna. The EM wave radiated from the antenna passes through a Spiral Phase Plate (SPP) to endow the wave with OAM. The SPP is mounted on a frame and can be rotated by a motor whose rotation speed is controllable. At the receiving side, two waveguide antennas are used to receive the signals from a partition of the OAM waves. The signals received by the two antennas are recorded by a high-speed sampling oscilloscope. These data are then exported to a computer and processed with MATLAB software. In MATLAB, a two-point interpolation algorithm based on Minimum Mean Square Error (MMSE) is used to combine the two signals received by the antennas. The interpolated signal is then exported to a spectrum analyser for spectrum display. Different rotational speeds of the interpolated antenna will result in different frequency shifts to be observed in the spectrum analyser.

Alignment of the experimental apparatus

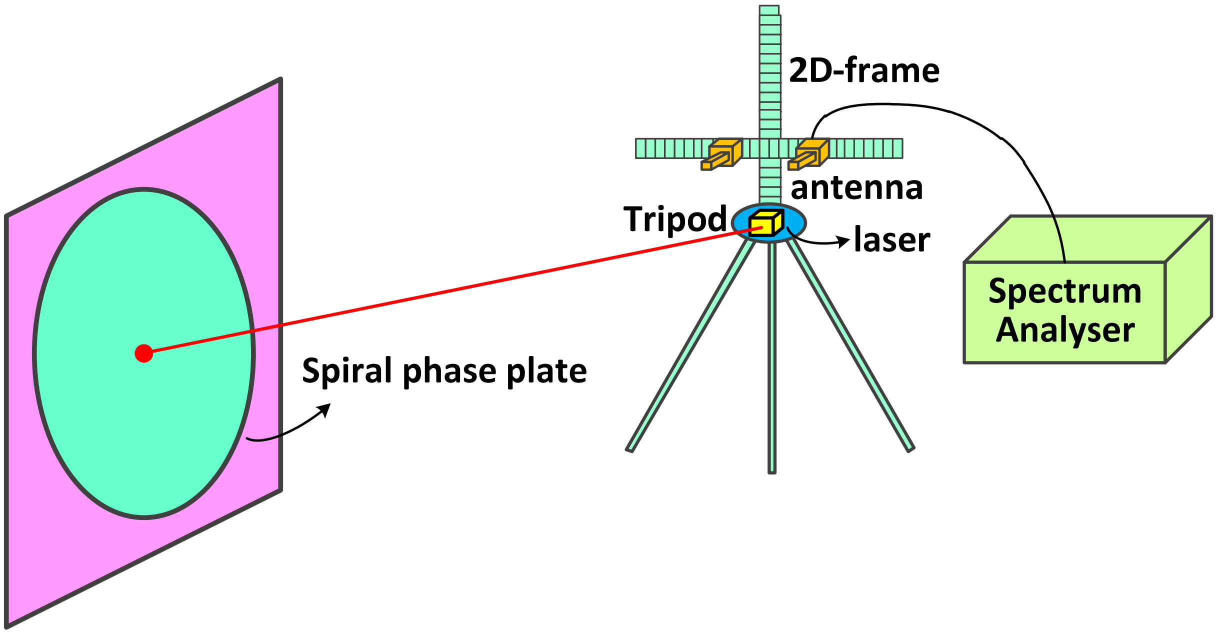

Figure|S2. Alignment of the experimental apparatus. The receiving antennas are mounted on a two-dimensional frame. A laser is mounted on the tripod for aligning with the SPP. Only when the laser beam irradiates the centre of the SPP will the receiving frame be aligned with the transmitting antenna. Then, the waveguide antennas are moved on the 2D frame. Only when the maximum energy is observed on the spectrum analyser, will the antennas be located on the OAM beam.

Relationship between the frequency shift, the bandwidth, and the transmission rate

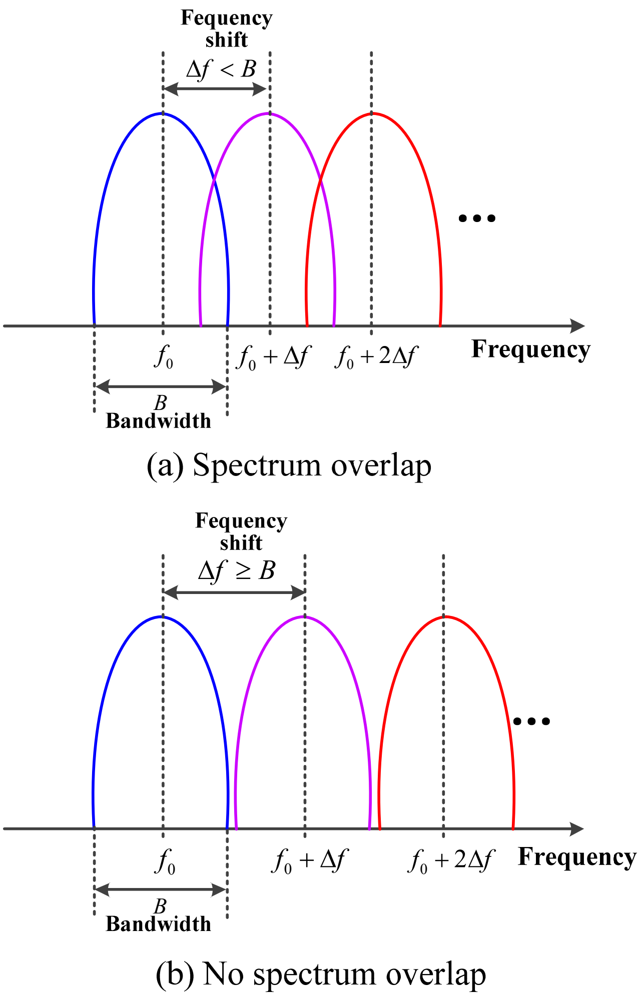

Figure|S3. Relationship between the frequency shift, the bandwidth and the transmission rate. (a) Spectrum overlap occurs when the frequency shift Δf is less than the bandwidth B. It creates interference in the spectrum and generates difficulty with the filtering process. Thus, in order to guarantee the quality of the spectrum, the bandwidth and the frequency shift must satisfy the relation $\Delta f \geq B$ **(b)** There is no spectrum overlap when the frequency shift Δf is larger than the bandwidth B. The relationship between the bandwidth and the transmission rate is given by the Shannon equation, i.e., $R_{\max} = B\log_2(1+\eta)$, where η is the Signal-to-Noise Ratio (SNR). This equation reveals that the transmission rate is proportional to the bandwidth.

Detecting the data encoded on the OAM

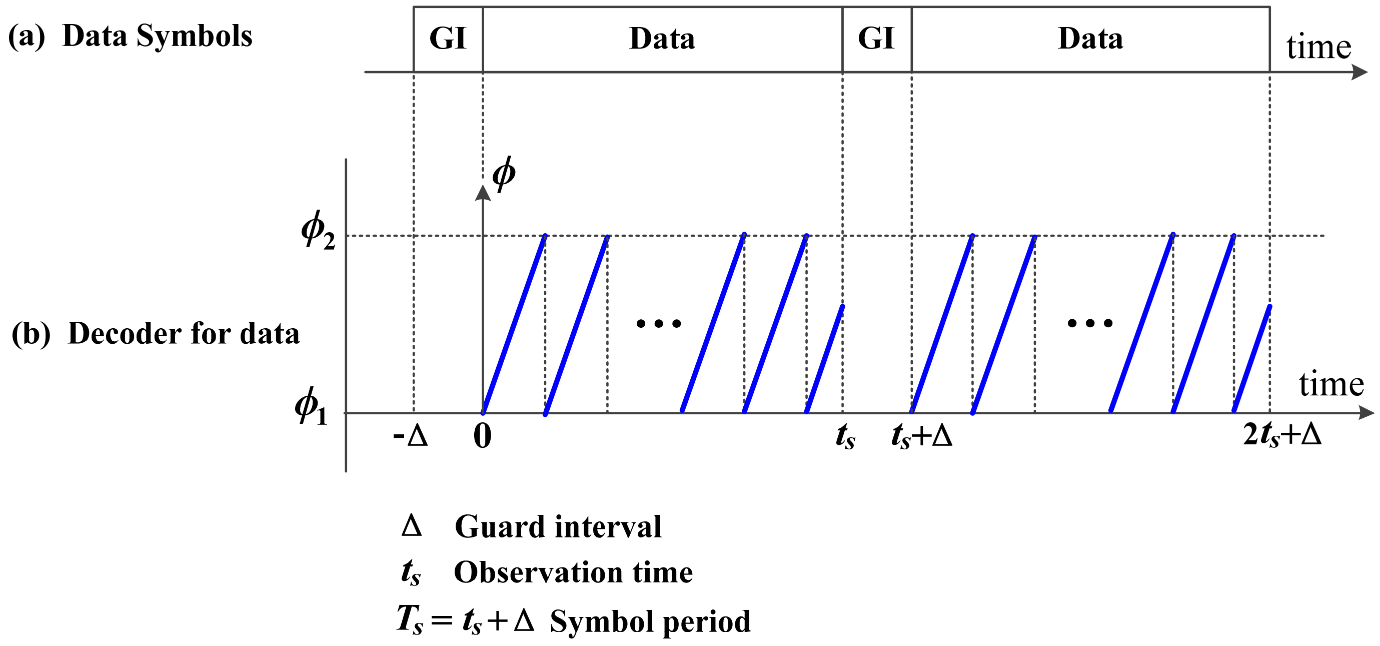

Figure|S4. Detecting the data encoded on the OAM. (a) Illustration of data symbols. GI: Guard Interval, Data: Effective Symbol Window. (**b)** Detecting the data encoded on the OAM by taking data symbols as an example. The position of the interpolated point for coherent detection of a data symbol is illustrated in the figure. To guarantee the integrity of data symbols, the position of the interpolated point should be within the observation time t_s in a symbol period. During the observation time, signals encoded on the OAM are received and decoded using the interpolation algorithm.

Interpolation based on antenna array

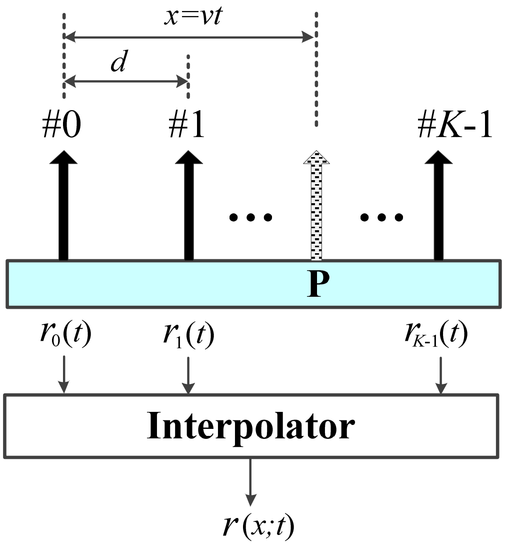

Figure|S5. Schematic of interpolation based on antenna array.

Assume that K antennas are employed for receiving the OAM signals. The received signals from the antennas can be expressed as

$$\mathbf{r}(t)=\left[r_0(t),\ r_1(t),\cdots,r_{K-1}(t)\right]^{\mathrm{T}} \tag{1}$$

The auto-correlation matrix and the cross-correlation matrix can be written as

$$\mathbf{R}(t)=E\left[\mathbf{r}(t)\cdot\mathbf{r}^{\mathrm{H}}(t)\right]=\left[\rho_{k,n}\right]\quad \mathbf{P}(x,t)=E\left[\mathbf{r}(t)\cdot r^{*}(x,t)\right]=\left[b_k(x)\right] \tag{2}$$

where "*" denotes conjugate.

According to the Ref. [19], the elements of the matrix can be derived as

$$\rho_{k,n}=J_0\left(2\pi d(n-k)/\tilde{\lambda}\right)\qquad b_k(x)=J_0\left(2\pi(kd-x)/\tilde{\lambda}\right) \tag{3}$$

where d is the antenna interval which can be written as $d = r\phi$, and r is the distance from the receiving to the propagation axis as shown in Fig. 2(a), ϕ is the angle between the antennas, $\tilde{\lambda}=2\pi r/l$ is the equivalent wavelength around the azimuth, $x=vt=2\pi\Omega rt$ is the position of the interpolated point P varying with time t and moving periodically between the position of antenna #0 and the position of antenna #K-1. Here, Ω is the rotational speed of the interpolated point. By substituting these parameters into Eq. (3), it can be derived that

$$\rho_{k,n}=J_0\left(l\phi(n-k)\right),\ b_k(x)=b_k(\Omega,t)=J_0\left(l(k\phi-2\pi\Omega t)\right) \tag{4}$$

Thus, the output of the interpolator can be expressed as

$$r_{\mathrm{VRA}}(\Omega,t)=\mathbf{w}^{\mathrm{T}}(\Omega,t)\mathbf{r}(t)=\mathbf{P}^{\mathrm{T}}(\Omega,t)\mathbf{R}(t)^{-\mathrm{T}}\mathbf{r}(t) \tag{5}$$

Verification of the generated OAM waves

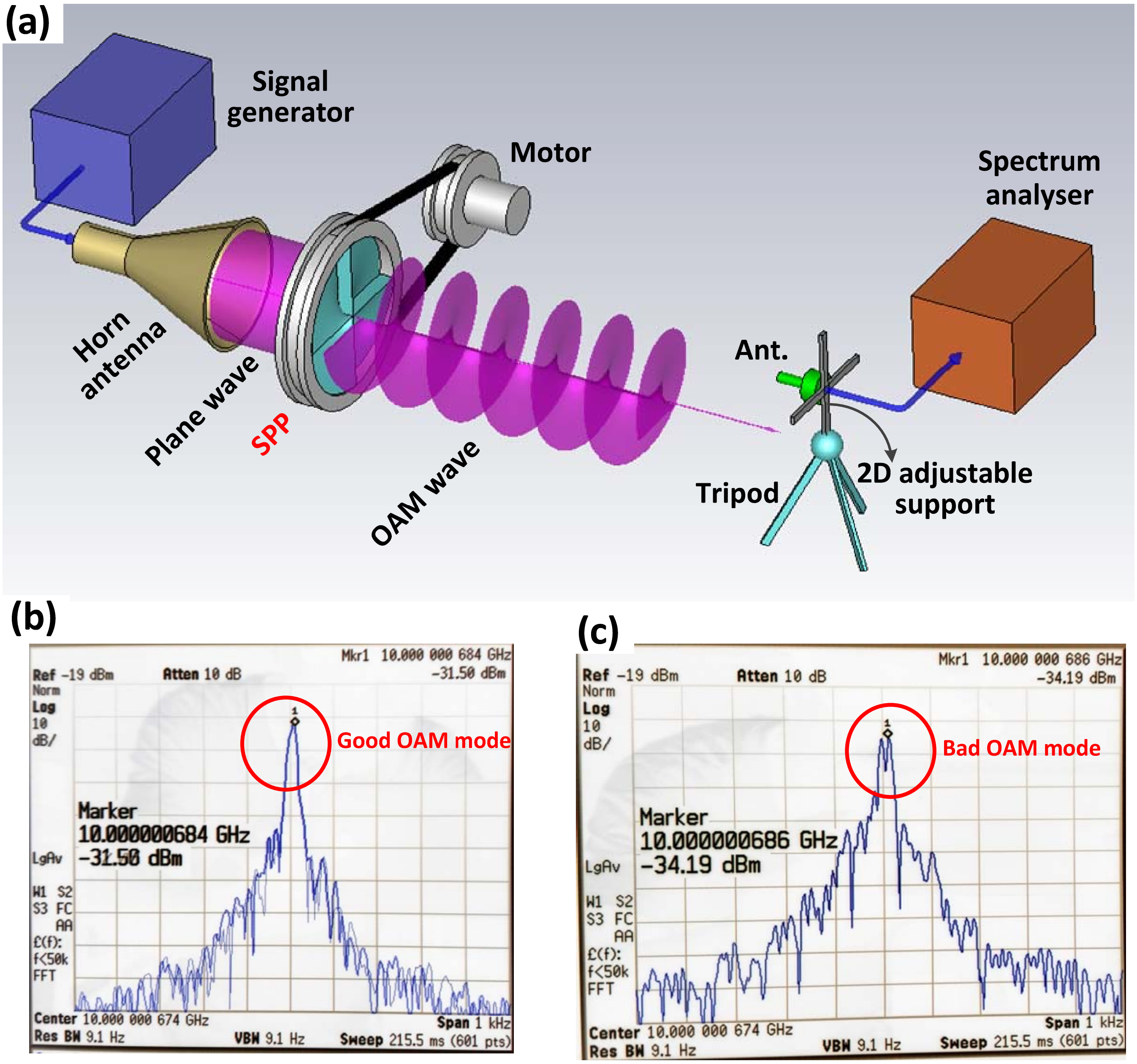

Figure|S6. Verification of the OAM wave. (a) Structure of the verification. To verify whether a good OAM wave is generated on the transmitting side, the SPP of OAM mode $l = 4$ is rotated with a speed of $r = 2.5$ (r/s) using a motor at the transmitting side, and a waveguide antenna is used to receive the signal and feed it to the spectrum analyser for observation. **(b,c)** Spectrum of the signal received from the rotational OAM wave. Only when a frequency shift of $\Delta f = l \times r$ (Hz) is observed by the spectrum analyser, would a good OAM wave have been generated at the transmitting side. As shown in this figure, a frequency shift of 10 Hz with respect to the centre frequency of 10.000000674 GHz is observed in Fig. S6(b). This revealed that a good OAM beam was generated at the transmitter. However, a bad spectrum will be observed if the transmitted OAM mode is not the expected one.

Observation of the signals

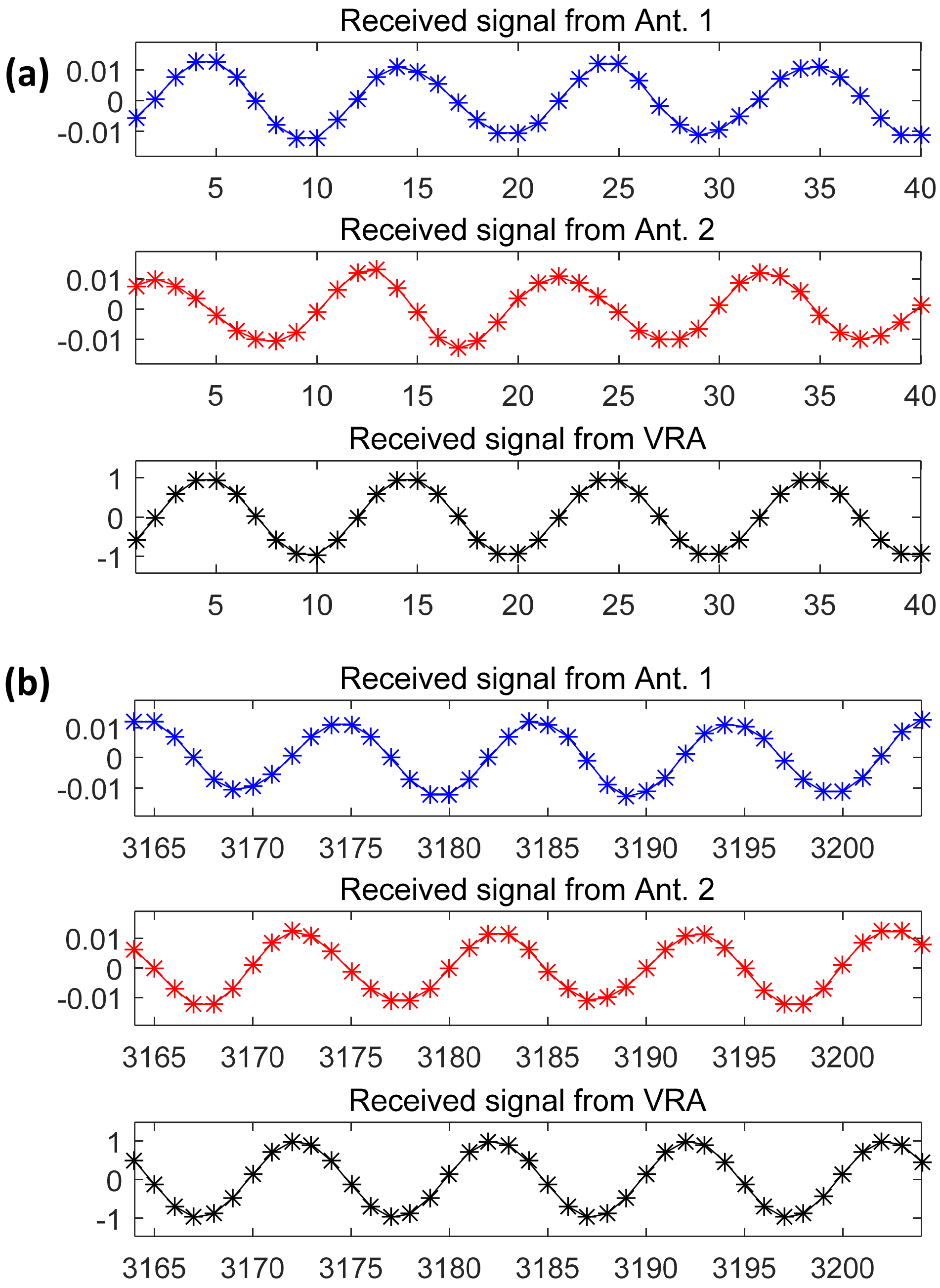

Figure|S7. Signals from the VRA. In the interpolation algorithm, a virtual antenna rotates between antenna 1 (Ant. 1) and antenna 2 (Ant. 2), which is produced by the interpolation algorithm based on the MMSE criterion. The frequency shift is proportional to the rotation speed Ω and the OAM mode. The signal from the Virtual Rotational Antenna (VRA) is the one that varies from Ant. 1 and Ant. 2 with the rotation speed. As shown in Fig. S7(a), at the beginning of interpolation, the virtual antenna is located at the position of Ant. 1, and the signal from the virtual antenna is the same as the one from Ant. 1. At the end of one interpolation period shown in Fig. S7(b), the virtual antenna is located at the position of Ant. 2, and the signal from the virtual antenna will be the same as the one from Ant. 2.

Data playback for the interpolated signal

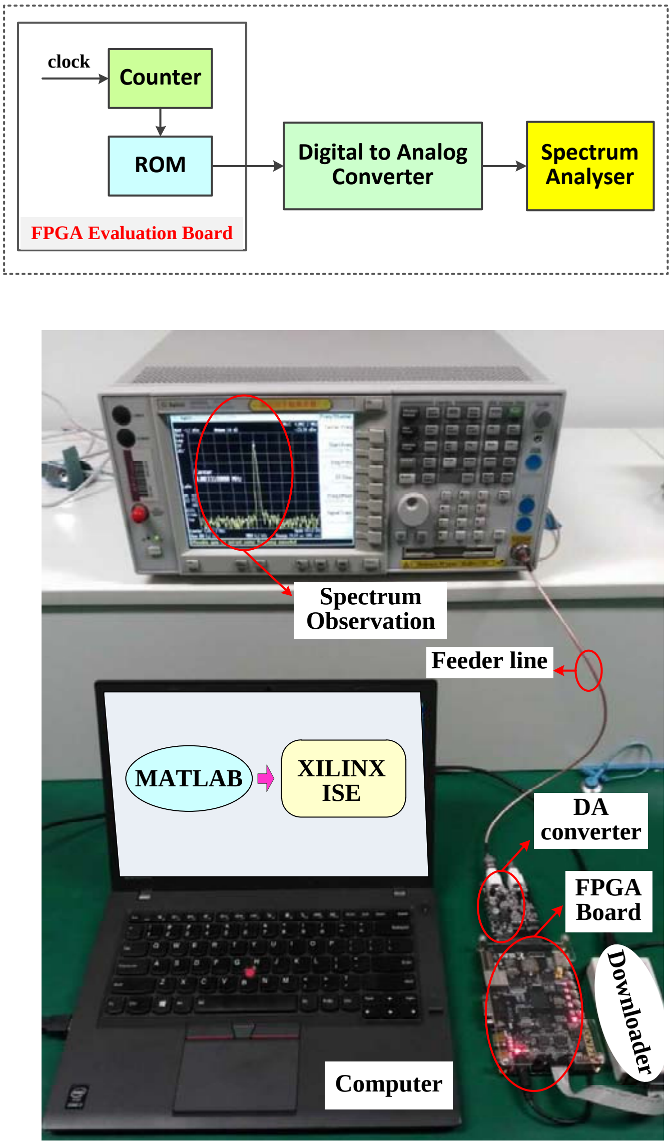

Figure|S8. Data playback of the interpolated signals. (a) Schematic of the data playback. The interpolated signals are first down-converted to an intermediate frequency of zero by mixing with a carrier signal of 10 GHz frequency, and are then filtered by a low-pass filter in the computer. Then, the down-converted signals are exported from the computer to an FPGA development board (Spartan-6 XC6SLX16-2CSG324C). In the FPGA board, the data exported from the computer are stored in Read Only Memory (ROM), and a counter is used for addressing the ROM consecutively. The digital data output from ROM is converted to an analogue signal by a Digital to Analogue Converter (DAC). Signals from the DAC are fed into the spectrum analyser (Agilent E4446A) to observe the spectrum. **(b)** Apparatus used for data playback.
